# Supplementary material for: Legacies of temperature fluctuations promote stability in marine biofilm communities
Source: Nat Commun. 2025 Mar 11;16:2442. doi: 10.1038/s41467-025-57258-y (PMC11897366; doi:10.1038/s41467-025-57258-y)
Supplement: Supplementary file 1 — Supplementary Information [file 41467_2025_57258_MOESM1_ESM.docx]

**Supplementary Information**

**Legacies of temperature fluctuations promote stability in marine biofilm communities**

Luca Rindi^1*^, Jianyu He^1,2^, Mara Miculan^3,4^, Matteo Dell’Acqua^3^, Mario Enrico Pè^3^, Lisandro Benedetti-Cecchi^1^

^1^Department of Biology, University of Pisa, Via Derna 1, Pisa, Italy

^2^Current address: Marine Science and Technology College, Zhejiang Ocean University, Zhoushan City, Zhejiang, China

^3^Institute of Plant Sciences, Scuola Superiore Sant’Anna, Pisa, Italia

^4^Current address: Center of Excellence for Sustainable Food Security, Biological and Environmental Sciences and Engineering Division (BESE), King Abdullah University of Science and Technology (KAUST), Thuwal, Saudi Arabia

*Corresponding author:

Luca Rindi

Department of Biology

University of Pisa

Via Derna 1

56126, Pisa, Italy

tel: +39050 2211415

fax: +39050 2211410

email: [luca.rindi@unipi.it](mailto:luca.rindi@unipi.it)

It includes Supplementary Figures 1-10.

**
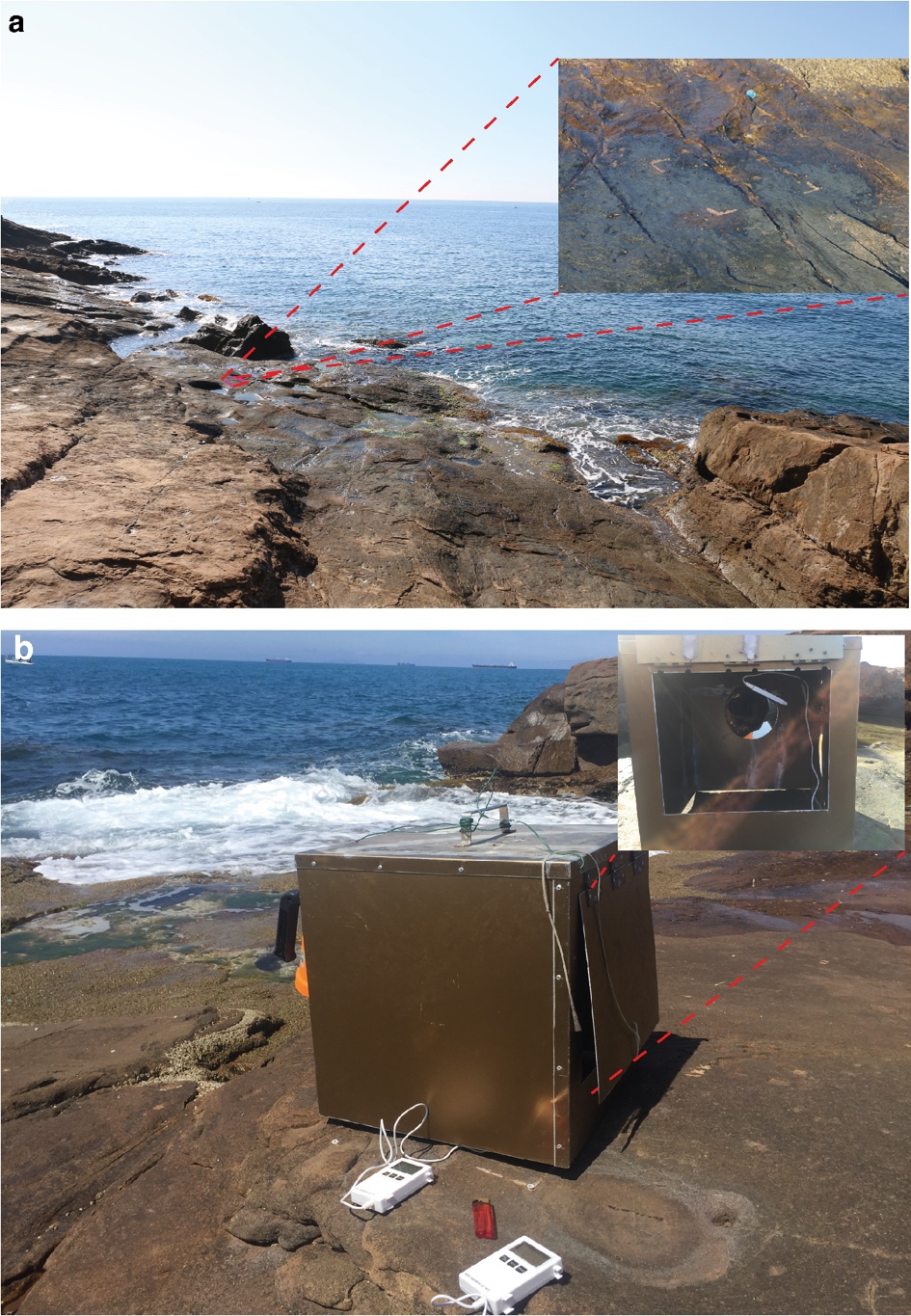
**

**Supplementary Figure 1**. Images of the study site and experimental treatments. **a**, Landscape view of the study site (June 2018). Areas occupied by the biofilm are darker than surrounding areas. The inset represents a close-up experimental unit (plot of 40 × 40 cm). **b**, Aluminium chamber equipped a with low-power butane heater. In the close-up, temperature loggers (button FT 800/System ©), which were used to record air inside the chambers continuously, are visible, together with a digital thermometer, which was used to the monitor the ambient and internal air temperature during the warming sessions.

**
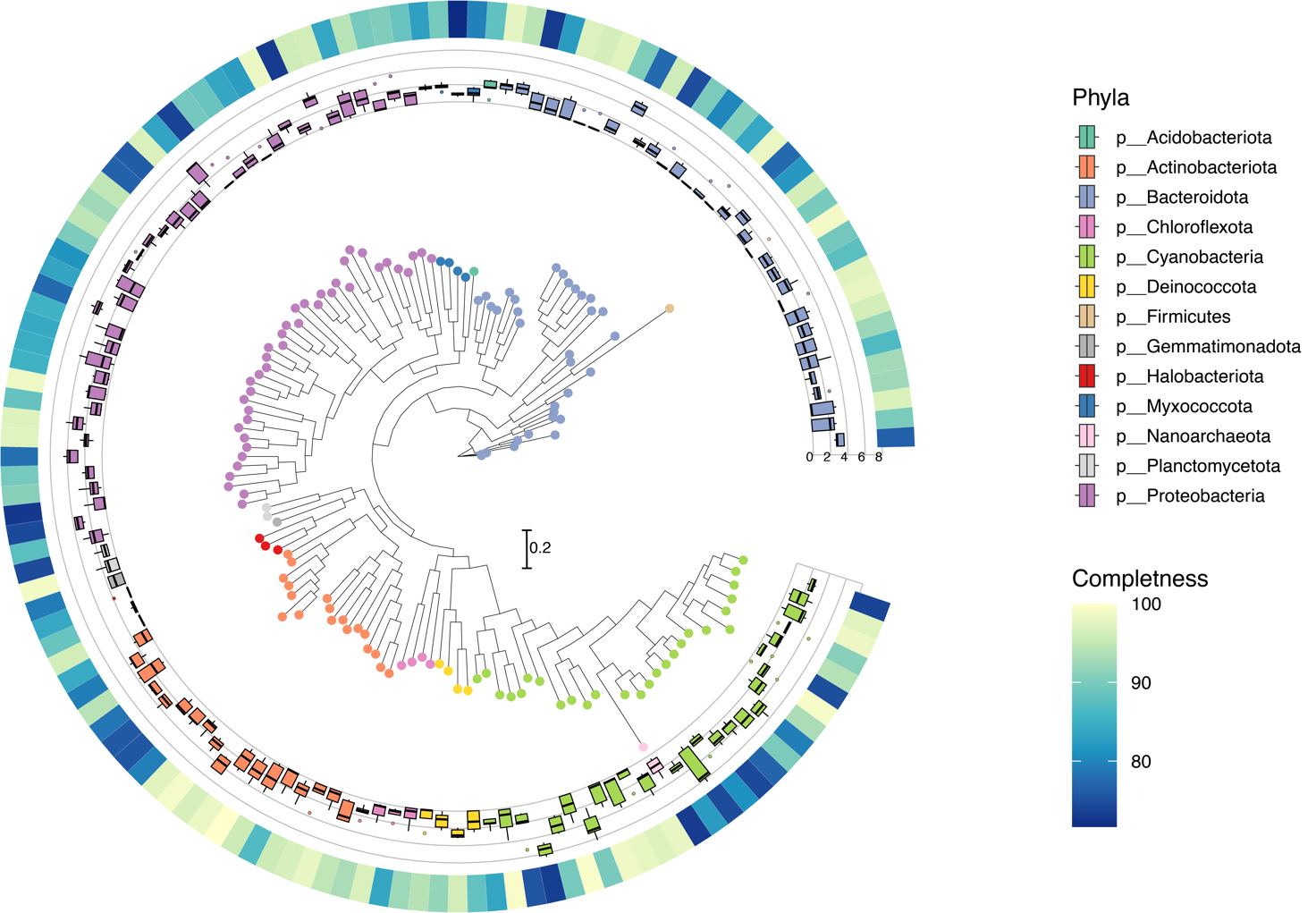
**

**Supplementary Figure 2**. Phylogenetic tree of 136 Metagenome-Assembled Genomes (MAGs) reconstructed from 77 samples collected throughout the experiment. MAGs are color-coded by phylum. From inside to outside, the rings display (i) a boxplot of MAG abundance (expressed as log(TPM + 1)) and (ii) the estimated genome completeness. One MAG was excluded from this analysis due to excessive gaps and poor alignment quality with markers, which could introduce artifacts in the phylogenetic inference. The phylogenetic tree is available in the online repositories (see the Data availability section).


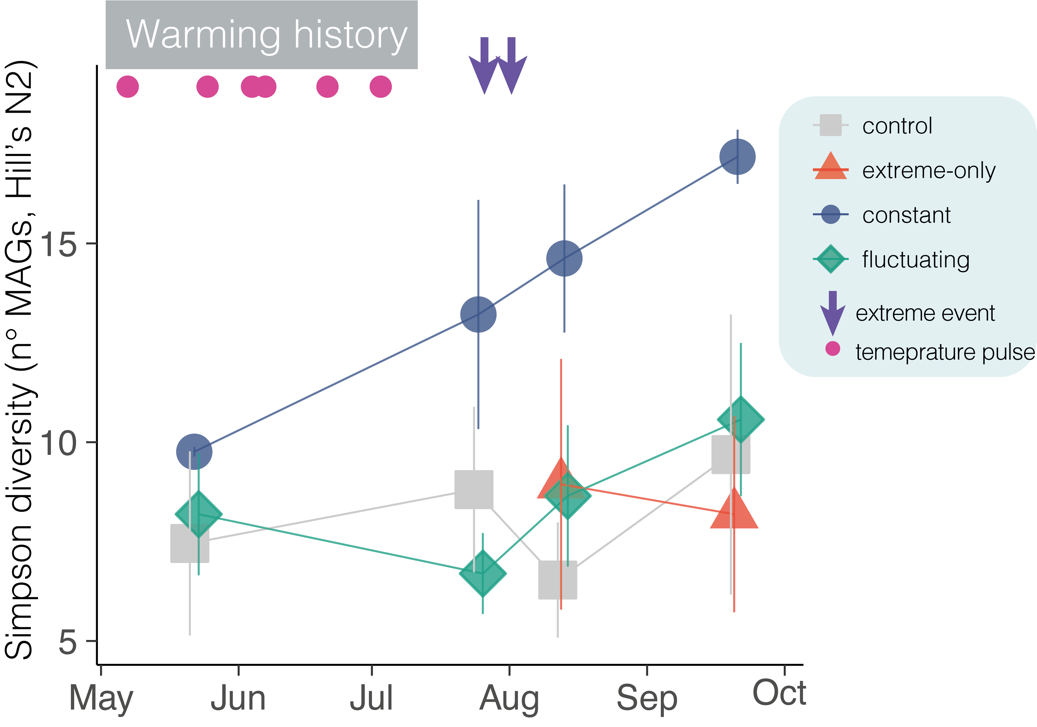


**Supplementary Figure 3**. Simpson diversity calculated as the equivalent number of species (Hill numbers) under different temporal regimes of warming. Pink circular circles indicate temperature pulses, while purple down-facing arrows indicate the extreme temperature events. Error-bars are the standard error of the mean (*n* = 3 for controls, fixed and extreme-only treatments and *n* = 9 for the fluctuating treatment). All statistical values are provided in Supplementary Table 1 (available in the Source Data).


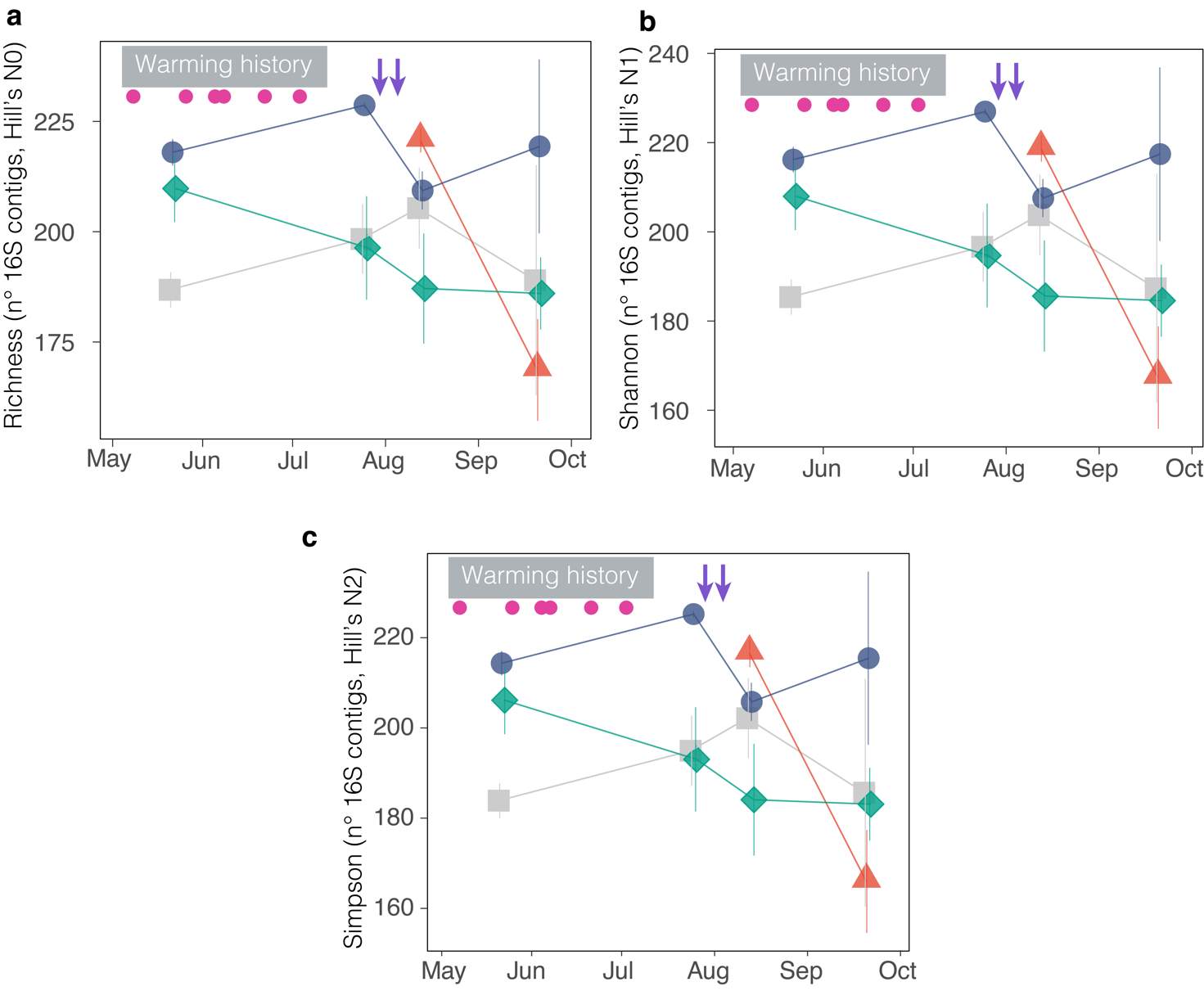


**Supplementary Figure 4. a**, Time-series of richness, **b**, (# 16S contigs), Shannon diversity (Hill number of order 1) **c**, Simpson diversity (Hill number of order 2). These diversity indices were calculated based on the reconstruction of the 16S rRNA gene (16S rRNA contigs) and its quantification across samples. Error bars indicate the standard errors of the mean (controls and extreme-only treatments, *n* = 3; fixed warming treatment, *n* = 2 at the first sampling and *n* = 3 thereafter). Prior to the imposition of extreme temperatures, the control condition represents the average of six plots (n = 6; three extreme-only and three control plots), while the fluctuating treatment is shown as the average of nine plots (*n* = 9) derived from three fluctuating sequences (fluct-s1, fluct-s2, and fluct-s3). Pink circles indicate temperature pulses, while purple down-facing arrows indicate the extreme temperature events. All statistical values are provided in Supplementary Table 2 (available in the Source Data). Source data are provided as a Source Data file.


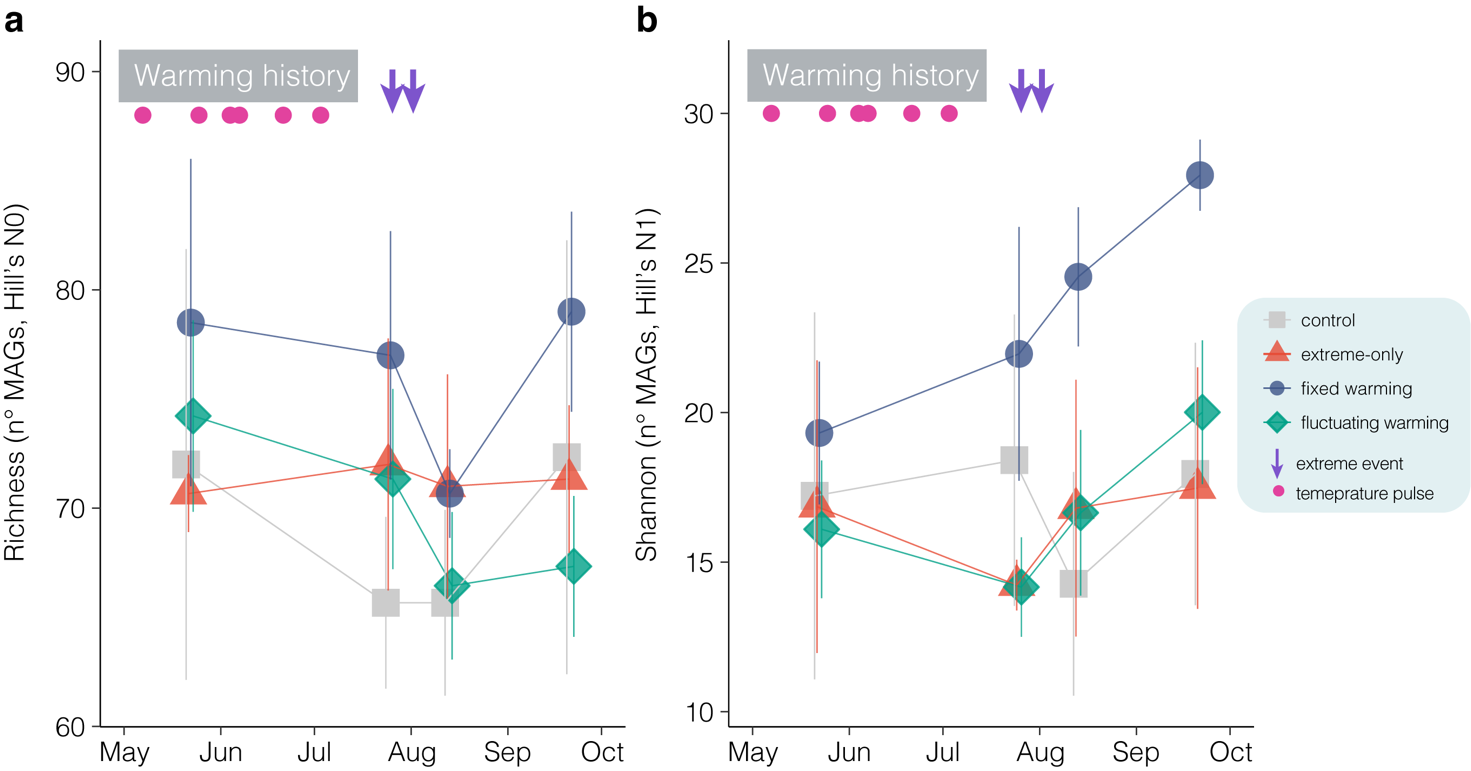


**Supplementary Figure 5**. Time series of **a**, richness and **b** Shannon diversity (Hill number of order 1) under different temporal regimes of warming. Control and extreme-only conditions were plotted separately throughout the entire duration of the study. Error bars represent standard errors of the mean (*n* = 3) for controls, fixed warming, and extreme-only treatments. The fluctuating treatment is shown as the average (*n* = 9) of three fluctuating sequences (fluct-s1, fluct-s2, and fluct-s3). Pink circles denote temperature pulses, while purple downward-facing arrows indicate the extreme temperature events. All statistical values are provided in Supplementary Table 3 (available in the Source Data). Source data are provided as a Source Data file.


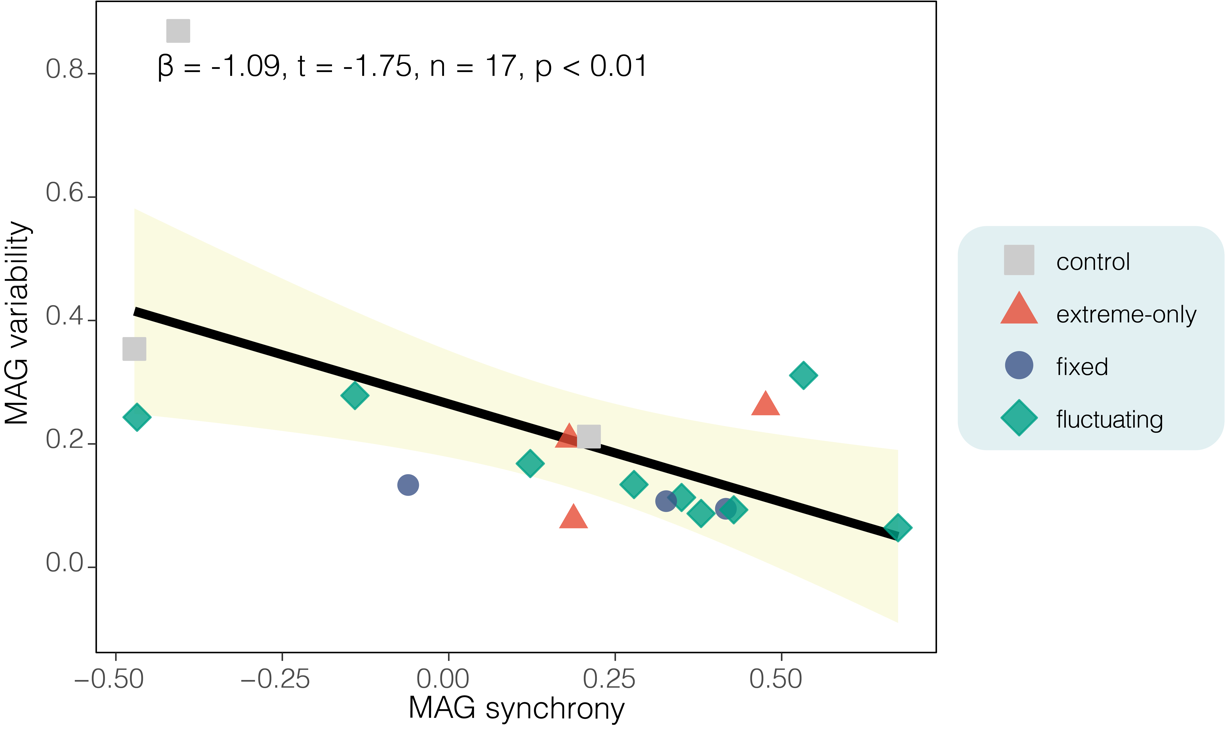


**Supplementary Figure 6. Negative relationship between MAG variability and MAG synchrony.** Source data are provided as a Source Data file.


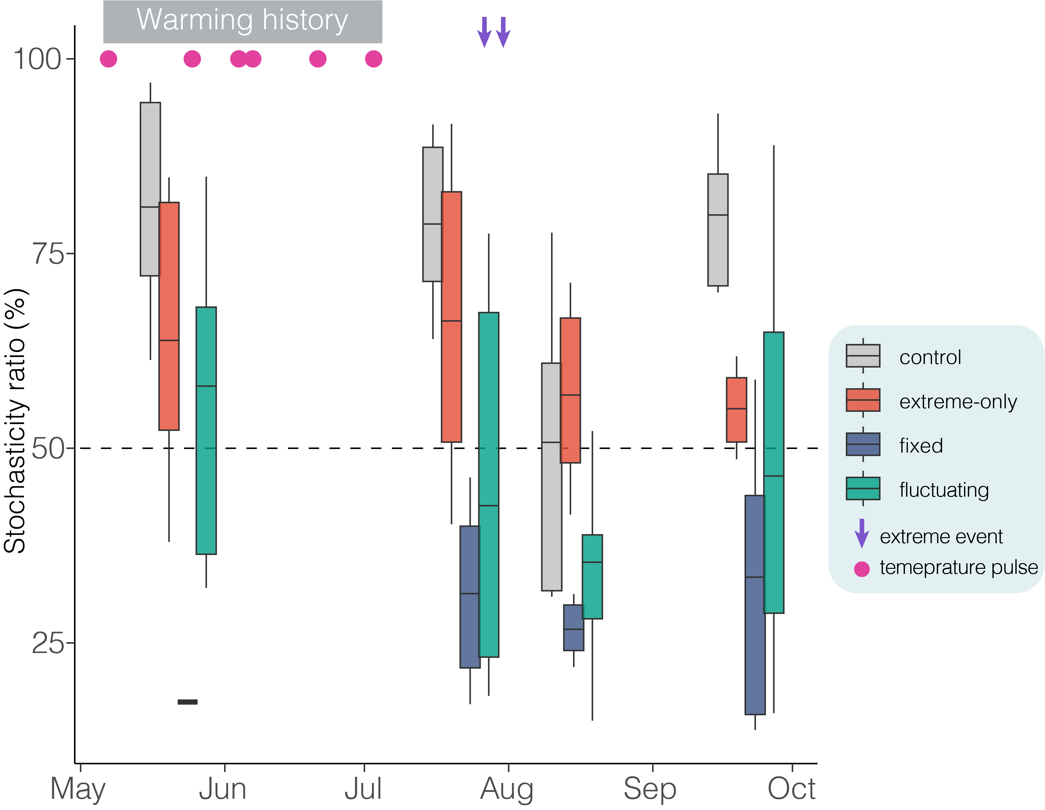


**Supplementary Figure 7**. Time-series of phylogenetic Normalized Stochasticity Ratio (NST) under different temporal regimes of warming. Control and extreme-only conditions were plotted separately throughout the entire duration of the study (*n* = 3). Fluctuating treatment is the average of plots (*n* = 9) of three fluctuating sequences (fluct-s1, fluct-s2 and fluct-s3). Values in the boxplot is the result of **n(n-1)/2 pairwise NST values**. The central line of boxplots represents the mean, the box spans the interquartile range (25^th^–75^th^ percentiles), whiskers extend to the 10^th^ and 90^th^ percentiles, and points indicate outliers. Pink circles indicate temperature pulses, while purple down-facing arrows indicate the extreme temperature events. All statistical values are provided in Supplementary Table 6 (available in the Source Data). Source data are provided as a Source Data file.


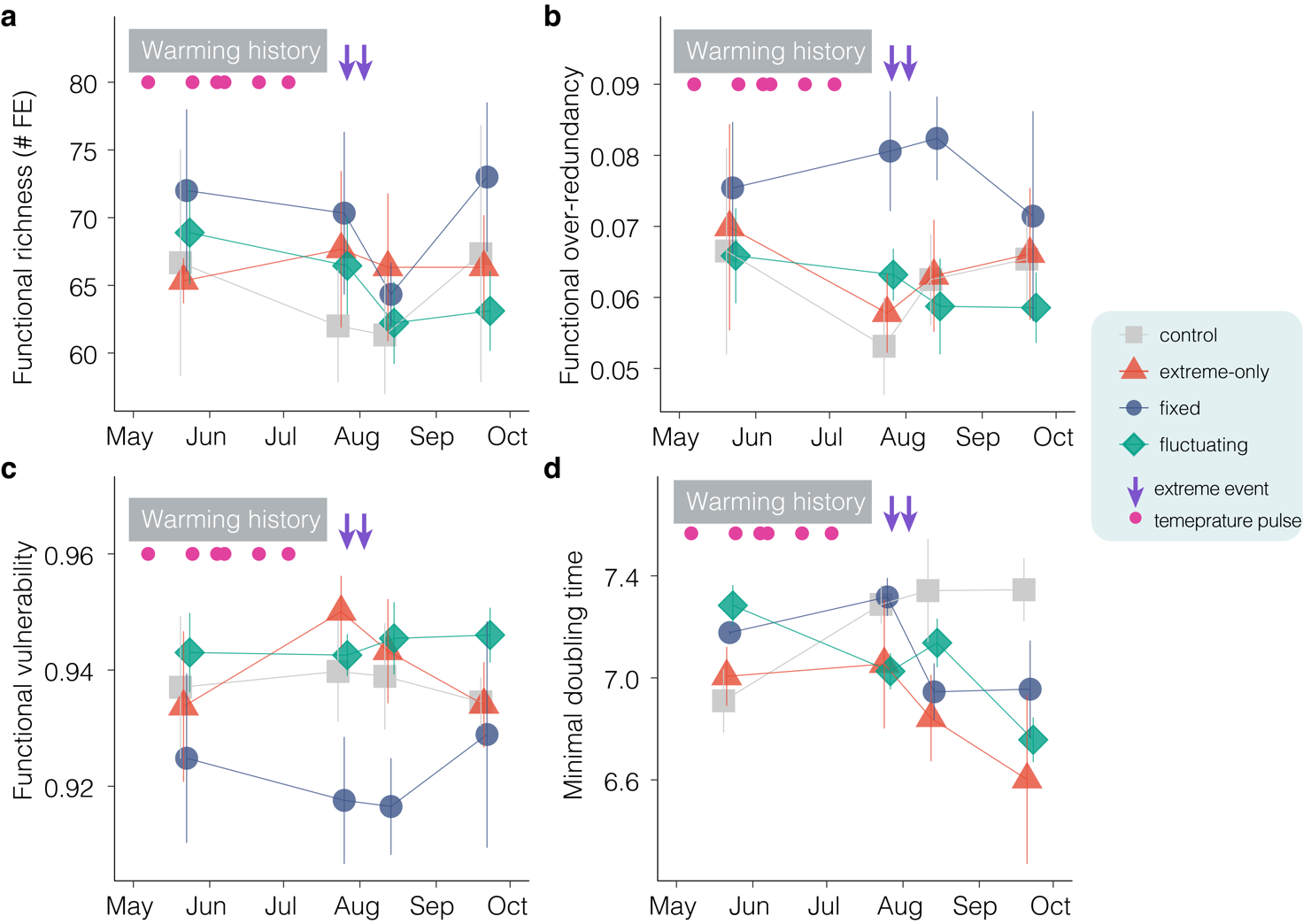


**Supplementary Figure 8**. **a**, Time series of functional group richness (FR), **b**, functional over-redundancy (FOR), **c**, functional vulnerability (FVuln) of stress-tolerance traits of biofilm community and **d**, Minimal Doubling Time. Error bars indicate the standard errors of the mean (controls and extreme-only treatments, n = 3; fixed warming treatment, n = 2 at the first sampling and n = 3 thereafter). Prior to the imposition of extreme temperatures, the control condition represents the average of six plots (n = 6; three extreme-only and three control plots), while the fluctuating treatment is shown as the average of nine plots (n = 9) derived from three fluctuating sequences (fluct-s1, fluct-s2, and fluct-s3). Pink circles denote temperature pulses, while purple downward-facing arrows indicate the extreme temperature events. Source data are provided as a Source Data file.


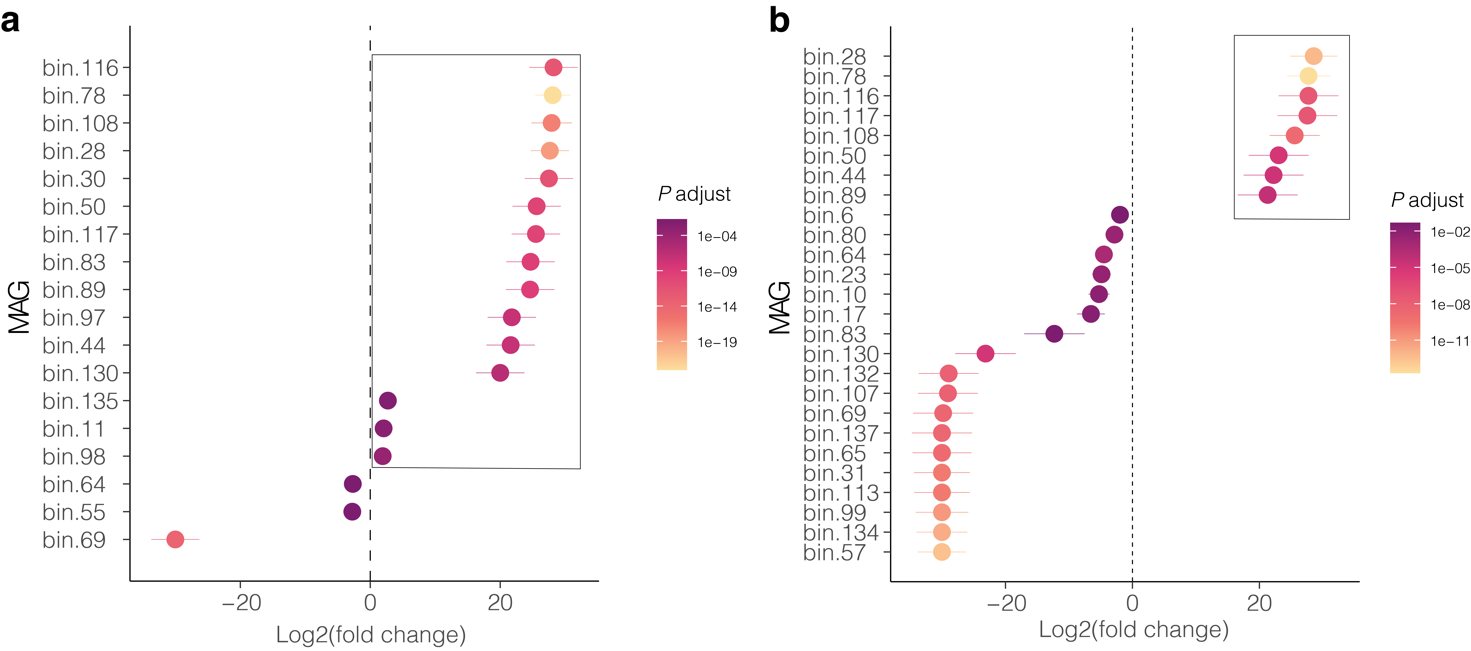


**Supplementary Figure 9.** Log₂-fold change of differentially abundant MAGs in fluctuating **a**, and fixed warming **b**, treatments compared to controls. Rectangles highlight MAGs with positive log₂-fold change values, indicating higher abundance relative to the control condition. P-values from the differential expression analysis were adjusted for multiple comparisons using the Benjamini & Hochberg method. Source data are provided as a Source Data file.


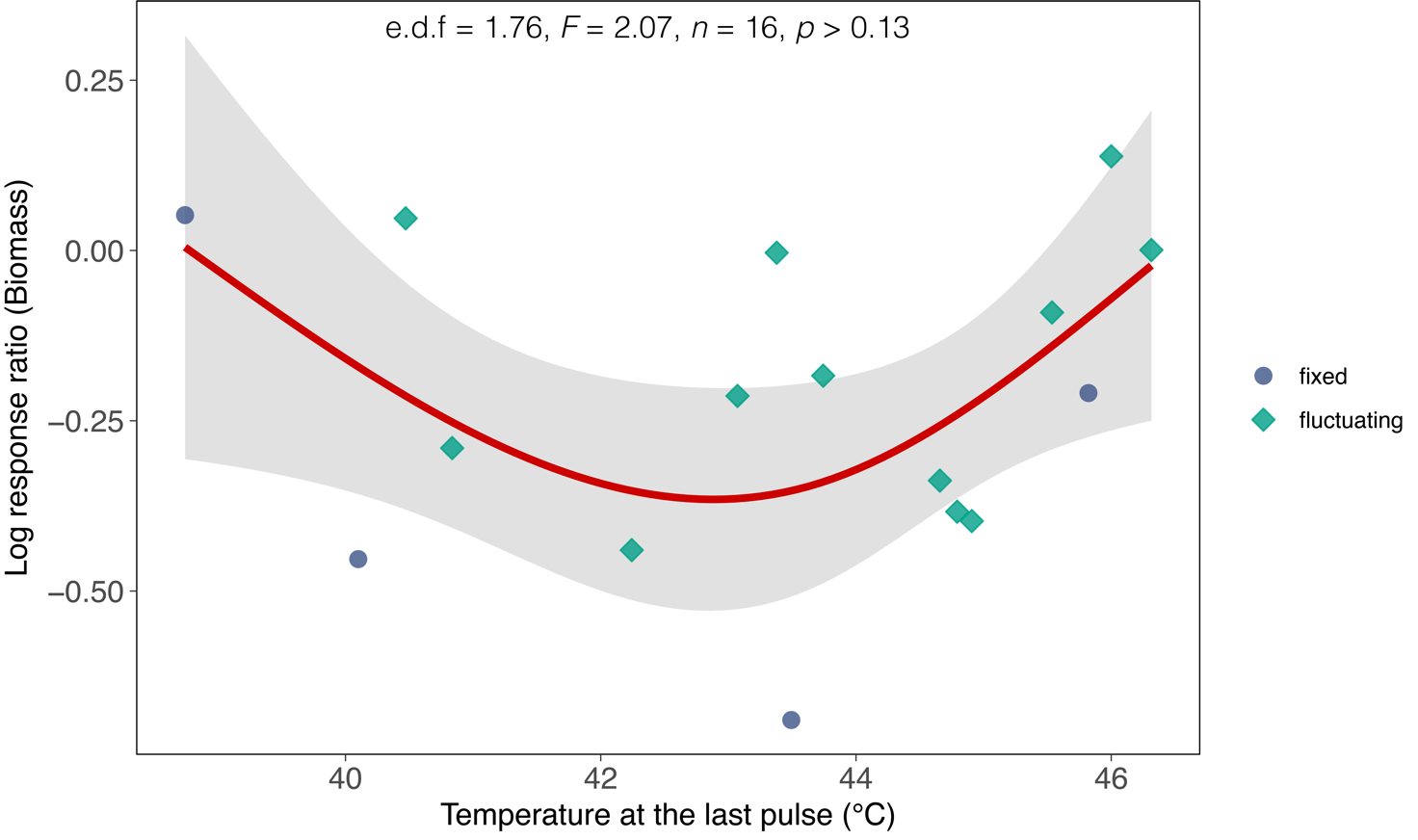


**Supplementary Figure 10.** A test for thermal acclimatization. If acclimatization was the main driver of biofilm response to extreme temperatures, we expected plots exposed to high temperatures at the last pulse event to be less impacted by extreme events. To test this prediction, we calculated changes in biofilm biomass that occurred between the imposition of the temperature extremes relative to that observed at the last warming pulse as log-response ratios and related these changes to the temperature at the last warming pulse using a generalized additive model (GAM). The lack of a significant relationships between these variables suggest acclimatization was not a relevant mechanism determining the sensitivity of biofilm to future extreme events. Source data are provided as a Source Data file.
