# Supplementary material for: Legacies of temperature fluctuations promote stability in marine biofilm communities
Source: Nat Commun. 2025 Mar 11;16:2442. doi: 10.1038/s41467-025-57258-y (PMC11897366; doi:10.1038/s41467-025-57258-y)
Supplement: Supplementary file 2 — Reporting Summary [file 41467_2025_57258_MOESM2_ESM.pdf]

nature portfolio

Corresponding author(s): Luca Rindi

Last updated by author(s): 03/02/2025

# Reporting Summary

Nature Portfolio wishes to improve the reproducibility of the work that we publish. This form provides structure for consistency and transparency in reporting. For further information on Nature Portfolio policies, see our Editorial Policies and the Editorial Policy Checklist.

## Statistics

For all statistical analyses, confirm that the following items are present in the figure legend, table legend, main text, or Methods section.

| n/a | Confirmed | |
|---|---|---|
| ☐ | ☒ | The exact sample size (*n*) for each experimental group/condition, given as a discrete number and unit of measurement |
| ☐ | ☒ | A statement on whether measurements were taken from distinct samples or whether the same sample was measured repeatedly |
| ☐ | ☒ | The statistical test(s) used AND whether they are one- or two-sided<br>*Only common tests should be described solely by name; describe more complex techniques in the Methods section.* |
| ☐ | ☒ | A description of all covariates tested |
| ☐ | ☒ | A description of any assumptions or corrections, such as tests of normality and adjustment for multiple comparisons |
| ☐ | ☒ | A full description of the statistical parameters including central tendency (e.g. means) or other basic estimates (e.g. regression coefficient) AND variation (e.g. standard deviation) or associated estimates of uncertainty (e.g. confidence intervals) |
| ☐ | ☒ | For null hypothesis testing, the test statistic (e.g. *F*, *t*, *r*) with confidence intervals, effect sizes, degrees of freedom and *P* value noted<br>*Give P values as exact values whenever suitable.* |
| ☒ | ☐ | For Bayesian analysis, information on the choice of priors and Markov chain Monte Carlo settings |
| ☐ | ☒ | For hierarchical and complex designs, identification of the appropriate level for tests and full reporting of outcomes |
| ☐ | ☒ | Estimates of effect sizes (e.g. Cohen's *d*, Pearson's *r*), indicating how they were calculated |

*Our web collection on statistics for biologists contains articles on many of the points above.*

## Software and code

Policy information about availability of computer code

| | |
|---|---|
| Data collection | ImageJ software (1.46) was utilized to determine Chlorophyll a concentration in the biofilm from multispectral digital images of the experimental plots. The codes are accessible on Figshare (DOI: 10.6084/m9.figshare.28319627.v1) |
| Data analysis | The software and R packages used in the analysis include MetaWRAP (version 1.3.0), along with its associated tools MEGAHIT (version 1.1.3), QUAST (version 5.0.2), and CheckM (version 1.0.12). The Genome Taxonomy Database (GTDB) used is release 95, and PhyloPhlAn is version 3.0. Functional and trait-based analyses were performed using microTrait (version 1.0), gRodon (version 1.0.4), and the Normalized Stochasticity Ratio (NST) tool (version 3.0.6). Functional diversity analysis utilized mFD (version 1.0.0). In R, statistical and visualization packages included dplyr (version 1.0.10), tidyr (version 1.2.1), ggplot2 (version 3.4.0), glmmTMB (version 1.1.4), and emmeans (version 1.8.4). |

For manuscripts utilizing custom algorithms or software that are central to the research but not yet described in published literature, software must be made available to editors and reviewers. We strongly encourage code deposition in a community repository (e.g. GitHub). See the Nature Portfolio guidelines for submitting code & software for further information.

## Data

Policy information about availability of data

All manuscripts must include a data availability statement. This statement should provide the following information, where applicable:

- Accession codes, unique identifiers, or web links for publicly available datasets
- A description of any restrictions on data availability
- For clinical datasets or third party data, please ensure that the statement adheres to our policy

All sequencing data are available in the NCBI BioProject PRJNA1062819 at: https://www.ncbi.nlm.nih.gov/bioproject/PRJNA1062819 . All other relevant data generated or analyzed during this study are included in the manuscript and supplementary information. Source data are available for Figs. 1c,d, 2, 3, 4, 5, 6b-d, and Supplementary Figs. 3–9 in the associated source data file. Source data are provided with this paper. Data that support the findings of this study can be accessed on Figshare (DOI: 10.6084/m9.figshare.28319627.v1).

## Research involving human participants, their data, or biological material

Policy information about studies with human participants or human data. See also policy information about sex, gender (identity/presentation), and sexual orientation and race, ethnicity and racism.

| | |
|---|---|
| Reporting on sex and gender | NA |
| Reporting on race, ethnicity, or other socially relevant groupings | NA |
| Population characteristics | NA |
| Recruitment | NA |
| Ethics oversight | NA |

Note that full information on the approval of the study protocol must also be provided in the manuscript.

# Field-specific reporting

Please select the one below that is the best fit for your research. If you are not sure, read the appropriate sections before making your selection.

☐ Life sciences ☐ Behavioural & social sciences ☒ Ecological, evolutionary & environmental sciences

For a reference copy of the document with all sections, see nature.com/documents/nr-reporting-summary-flat.pdf

# Ecological, evolutionary & environmental sciences study design

All studies must disclose on these points even when the disclosure is negative.

| | |
|---|---|
| Study description | A two-phase field experiment was performed to assess how warming history shaped the structure of the biofilm community and its multiple dimensions of stability in response to subsequent perturbations. The treatments included a control with natural temperature variation, fixed warming with repeated 12°C pulses above ambient temperature, and fluctuating warming with varied pulses that maintained the same mean temperature. To generalize the effect of fluctuating warming and avoid reliance on a single sequence, we established three distinct temporal warming sequences (fluct-s1, fluct-s2, and fluct-s3) that shared the same mean (ΔT = 12 °C) and variance (SD = 5 °C) but differed in their temporal profiles. After the initial warming treatments, a severe thermal shock of 60 °C for 120 minutes was applied twice over four days. This extreme temperature, relevant to occasional site conditions, was also tested on four additional plots that had not been pre-exposed to warming (Extreme-Only). Heating was conducted using aluminum chambers with stoves, and aerial temperatures were monitored with iButton loggers placed both inside and outside the chambers. To control for shading effects during heating, four plots were shaded but not warmed, and four unaltered plots served as overall controls. |
| Research sample | We established a total of 28 plots (over 2 km area), each consisting of a 40x40 cm area of substrate covered with biofilm (community of cyanobacteria and microalgae living embedded in a matrix of self-produced polymeric substances). These plots were spaced 2-8 meters apart to ensure for spatial indipendence. While acknowledging the spatial and temporal variability of rocky intertidal biofilm, we reference previous studies that successfully discerned the effects of warming using 3 to 4 replicate plots within the same study site (Dal Bello et al. 2015, Rindi et al. 2022). Based on this precedent, we believe that our sample size is sufficient. It provides the necessary precision to distinctively separate the effects of different warming regimes. This approach aligns with established methodologies in our field, ensuring that our findings are both reliable and scientifically valid. |
| Sampling strategy | Biofilm biomass was evaluated for experimental plots in 9 times during the durations of the study, except for Extreme-only plots that were sampled starting from mid-June, 40 days before the imposition of temperature extremes (LR and HJ). Biofilm communities were sequenced at four key stages: the experiment's start, post-thermal history imposition, after extreme events, and at the experiment's conclusion (LR and JH). This was conducted across 3 plots per treatment, resulting in a total of 78 samples. |

| | |
|---|---|
| Data collection | Rock samples covered  in biofilm were collected at four different times during the experiment, using a hammer and chisel, from three plots per treatment, yielding 78 samples in total (LR and HJ). Samples were taken randomly, with a 5 cm buffer from adjacent sample edges, and stored under cold, dark conditions before freezing at -80 °C. For DNA extraction, excess rock was manually removed, samples were pulverized, and DNA was extracted using the Qiagen DNeasy PowerSoil Kit (HJ). DNA quality was assessed using NanoDrop and Qubit systems (HJ). Sequencing was performed on an Illumina HiSeq2500, with reads de-multiplexed based on the Illumina indexing system. |
| Timing and spatial scale | The study was performed between May and September 2018. Warming pulses were performed for six times at biweekly frequency from May to August 2018 (2018-05-07, 2018-05-25, 2018-06-04, 2018-06-07, 2018-06-21, 2018-07-03)(LR and JH). Extreme temperature treatments were applied on two days—approximately one week after the imposition of warming pulses—on 2018-07-26 and 2018-07-30. Biofilm biomass was evaluated using an IR sensing camera on nine dates throughout the study (2018-05-22, 2018-05-30, 2018-06-20, 2018-06-27, 2018-07-09, 2018-08-13, 2018-08-29, 2018-09-10, and 2018-09-21). Note that Extreme-only plots were sampled starting in mid-June, 40 days before the imposition of temperature extremes.Rock samples covered in biofilm for DNA extraction were collected at four different times (2018-05-22, 2018-07-25, 2018-08-13, 2018-09-21). |
| Data exclusions | All biofilm biomass data were included in the analysis. However, for DNA analysis, one rock sample from the fixed-warming treatment, collected during the first sampling, was excluded from sequencing due to its excessively low DNA concentration, resulting in a total of 77 samples. |
| Reproducibility | We used four replicate plots per experimental condition for biomass measurements to ensure robust statistical power and reproducibility. To further validate our findings, we established three distinct random temporal warming sequences. Each sequence maintained the same mean and variance, ensuring that the overall intensity and variability of warming were controlled, while differences in their temporal profiles allowed us to demonstrate that the observed effects of fluctuating warming were not specific to a single pattern. This dual approach—adequate replication coupled with variability in the warming sequences—confirms that our results are both reliable and generalizable, strengthening the overall robustness of our experimental design. |
| Randomization | Experimental plots were randomly allocated to experimental treatements. |
| Blinding | The process of data collection and analysis is blind before relevant results are revealed. |

Did the study involve field work? ☒ Yes ☐ No

## Field work, collection and transport

| | |
|---|---|
| Field conditions | The study was performed along rocky shore of Calafuria (Livorno, 43° 30'N, 10°19' E) between May and September 2018. The coast is composed of gently sloping sandstone platforms with high-shore levels (0.3 - 0.5 m above mean low-level water) characterized by assemblages of barnacles interspersed among areas of seemingly bare rock, where the biofilm develops. The annual average temperature of the study site is around 16.4 °C, and temperature during the study period (May-September) spans a range from about 18.2 °C to 24.8 °C. The average annual precipitation is approximately 953 mm and spans over range from about 62 mmin May to 112 mm in September. |
| Location | The study was performed along rocky shore of Calafuria (Livorno, 43° 30'N, 10°19' E). |
| Access & import/export | Rock samples were collected in compliance with national and local law. |
| Disturbance | No disturbance was caused. |

# Reporting for specific materials, systems and methods

We require information from authors about some types of materials, experimental systems and methods used in many studies. Here, indicate whether each material, system or method listed is relevant to your study. If you are not sure if a list item applies to your research, read the appropriate section before selecting a response.

## Materials & experimental systems

| n/a | Involved in the study | |
|---|---|---|
| ☒ | ☐ | Antibodies |
| ☒ | ☐ | Eukaryotic cell lines |
| ☒ | ☐ | Palaeontology and archaeology |
| ☒ | ☐ | Animals and other organisms |
| ☒ | ☐ | Clinical data |
| ☒ | ☐ | Dual use research of concern |
| ☒ | ☐ | Plants |

## Methods

| n/a | Involved in the study | |
|---|---|---|
| ☒ | ☐ | ChIP-seq |
| ☒ | ☐ | Flow cytometry |
| ☒ | ☐ | MRI-based neuroimaging |

## Plants

Seed stocks

*Report on the source of all seed stocks or other plant material used. If applicable, state the seed stock centre and catalogue number. If plant specimens were collected from the field, describe the collection location, date and sampling procedures.*

Novel plant genotypes

*Describe the methods by which all novel plant genotypes were produced. This includes those generated by transgenic approaches, gene editing, chemical/radiation-based mutagenesis and hybridization. For transgenic lines, describe the transformation method, the number of independent lines analyzed and the generation upon which experiments were performed. For gene-edited lines, describe the editor used, the endogenous sequence targeted for editing, the targeting guide RNA sequence (if applicable) and how the editor was applied.*

Authentication

*Describe any authentication procedures for each seed stock used or novel genotype generated. Describe any experiments used to assess the effect of a mutation and, where applicable, how potential secondary effects (e.g. second site T-DNA insertions, mosiacism, off-target gene editing) were examined.*
